# Syntactic Computations in the Language Network: Characterizing Dynamic Network Properties Using Representational Similarity Analysis

**DOI:** 10.3389/fpsyg.2013.00271

**Published:** 2013-05-17

**Authors:** Lorraine K. Tyler, Teresa P. L. Cheung, Barry J. Devereux, Alex Clarke

**Affiliations:** ^1^Department of Psychology, Centre for Speech, Language and the Brain, University of CambridgeCambridge, UK

**Keywords:** syntax, sentence processing, syntactic ambiguity, language networks, magnetoencephalography, representational similarity analysis

## Abstract

The core human capacity of syntactic analysis involves a left hemisphere network involving left inferior frontal gyrus (LIFG) and posterior middle temporal gyrus (LMTG) and the anatomical connections between them. Here we use magnetoencephalography (MEG) to determine the spatio-temporal properties of syntactic computations in this network. Listeners heard spoken sentences containing a local syntactic ambiguity (e.g., “… landing planes …”), at the offset of which they heard a disambiguating verb and decided whether it was an acceptable/unacceptable continuation of the sentence. We charted the time-course of processing and resolving syntactic ambiguity by measuring MEG responses from the onset of each word in the ambiguous phrase and the disambiguating word. We used representational similarity analysis (RSA) to characterize syntactic information represented in the LIFG and left posterior middle temporal gyrus (LpMTG) over time and to investigate their relationship to each other. Testing a variety of lexico-syntactic and ambiguity models against the MEG data, our results suggest early lexico-syntactic responses in the LpMTG and later effects of ambiguity in the LIFG, pointing to a clear differentiation in the functional roles of these two regions. Our results suggest the LpMTG represents and transmits lexical information to the LIFG, which responds to and resolves the ambiguity.

## Introduction

Over the last 150 years substantial efforts have been made to understand the brain bases of human language. What this research has shown is that language function is instantiated in a bilateral fronto-temporal-parietal system, with different regions and combinations of regions within this system involved in different aspects of language. However, there is little agreement on the details of how different aspects of language are represented and processed within this neural system (Grodzinsky, 2000; Friederici et al., 2003; Hagoort, 2005; Tyler et al., 2011). Recent attempts to integrate these disparate findings into a coherent framework have placed renewed emphasis on the bi-hemispheric foundations of human language, taking into account data on the neurobiology of auditory processing in non-human primates and human studies on brain and language (Rauschecker and Tian, 2000; Jung-Beeman, 2005; Tyler and Marslen-Wilson, 2008; Bozic et al., 2010).

This bi-hemispheric model claims that human language is subserved by two main processing networks: one involving a bilateral temporal-parietal system which supports the semantic/pragmatic aspects of language, and a second left hemisphere (LH) fronto-temporal system which supports syntactic computations (Tyler and Marslen-Wilson, 2008). Human neuropsychological and neuroimaging evidence for this dual processing model comes from a variety of sources. For example, a number of studies have revealed a marked hemispheric asymmetry in favor of the LH in both fronto-temporal regions and in the white matter connections between them (Parker et al., 2005), providing the basis for a more functionally specialized LH system. Fronto-temporal regions in the LH have been consistently associated with syntactic analysis, although the specific frontal and temporal regions vary across studies. Moreover, recent experiments have established that the integrity of the LH fronto-temporal system, and of the connecting white matter tracts, is essential for syntactic analysis while RH fronto-temporal homologs are unable to take over this key linguistic function (Tyler et al., 2010; Papoutsi et al., 2011; Griffiths et al., 2013). In addition, the arcuate fasciculus, one of the direct fronto-temporal connecting white matter tracts, is not well-established either in non-human primates (Rilling et al., 2008) or in young children (Brauer et al., 2011), neither of which have well-developed syntactic capacities.

In contrast, mapping spoken inputs onto semantic representations and constructing semantic/pragmatic interpretations involves bilateral superior/middle temporal regions (Binder et al., 1997; Crinion et al., 2003; Scott and Wise, 2004; Tyler et al., 2010). Brain-damaged patients with extensive LH perisylvian lesions can still understand the meaning and pragmatic implications of spoken language, suggesting that these aspects of language function are subserved by a bilateral temporal system, with the RH able to assume adequate functionality in the absence of contributions from the LH (Longworth et al., 2005; Wright et al., 2011).

Under certain processing conditions these two components of the bi-hemispheric language system may be complemented by the contribution of other systems that subserve general cognitive processing demands, such as processes of selection and competition involving bilateral inferior frontal cortices (Thompson-Schill et al., 1997; Badre and Wagner, 2004; Bilenko et al., 2008; Bozic et al., 2010; Zhuang et al., 2011). Not all linguistic computations involve these general purpose systems, only those in which non-linguistic processing demands of various sorts are high.

Within this general framework an important goal is to be able to characterize the properties of the networks involved in language function. Toward this end we focus here on the computational properties of the LH fronto-temporal system, exploring the types of syntactic computations that it supports during spoken language comprehension. Many studies investigating the brain bases of syntactic analyses have implicated regions of the L inferior frontal cortex, BA 44 and/or 45, and the temporal cortex, either superior temporal gyrus or middle temporal gyrus (MTG) (Friederici et al., 2003; Snijders et al., 2009). In our own research, we have consistently found that L BA 45 and left posterior middle temporal gyrus (LpMTG) are implicated in syntactic analysis, together with the white matter tracts that directly connect them – the arcuate fasciculus and the extreme capsule fiber bundles. Perhaps the strongest evidence for the essential contribution of L BA45 and LpMTG to syntactic processing comes from studies combining functional and structural neuroimaging data with measures of syntactic performance in chronic stroke patients with LH damage. These enable us to draw strong inferences about the brain regions that are essential for the performance of a given neurocognitive process (Chatterjee, 2005; Fellows et al., 2005; Price et al., 2006). In our studies with patients, we find that syntactic deficits result from damage to either the left inferior frontal gyrus (LIFG; primarily BA 45), LpMTG (Tyler et al., 2010, 2011) or to disrupted functional or anatomical connectivity between them (Papoutsi et al., 2011; Rolheiser et al., 2011; Griffiths et al., 2013), establishing the importance of interactivity between LIFG and LpMTG during syntactic analysis. However, little is known about the types of syntactic computations subserved by the LIFG and LpMTG, how they are distributed over time across these regions, the relationship between them, or to what extent LIFG and LMTG play different roles in the processing of syntax.

Our starting point for investigating the neural computations involved in syntactic analysis is the claim that the phonological properties of spoken words activate their semantic and syntactic properties, which are assessed and integrated into the existing contextual representation (Marslen-Wilson and Tyler, 1980). This claim is supported by behavioral studies showing the early activation of different lexical properties and their on-line integration into the developing sentential representation (Marslen-Wilson et al., 1988; Zwitserlood, 1989). Neural signatures of lexical activation were initially revealed in ERP studies that found different types of neural response elicited by a variety of syntactic manipulations. The most robust finding is the P600, a positive response to syntactic manipulations at approximately 600 ms triggered by ungrammatical or non-preferred continuations of sentence fragments (Hagoort et al., 1993; Osterhout and Holcomb, 1993), and by ambiguity resolution (Kaan and Swaab, 2003). Other effects include an early left-anterior negativity (ELAN) after 150–200 ms (Hahne and Friederici, 1999; Friederici and Alter, 2004) elicited by violations of word category (Lau et al., 2006), and a subsequent (300–500 ms) left-anterior negative effect in response to morphosyntactic violations (Neville et al., 1991). However, since EEG has limited spatial resolution, these effects have only been broadly differentiated across the scalp.

In the present study we use magnetoencephalography (MEG) to ask how the activation of syntactic information and its integration into the developing sentential representation is distributed over time across the left fronto-temporal language system. We use syntactic ambiguity, rather than anomalies or violations, because syntactic ambiguity is an aspect of language processing that occurs naturally and frequently and does not involve ungrammaticality, in case ungrammaticality and violations induce additional processes not typically observed in normal on-line comprehension. We present listeners with spoken phrases which can be locally syntactically ambiguous (referred to here as the *central phrase*; e.g., “*juggling knives*”), heard in a sentential context (“In the circus, juggling knives ….”). The phrases are syntactically ambiguous between different syntactic roles; they can either be interpreted as a noun-phrase which functions as the subject of the embedded clause, or as a verb phrase in which the verb “juggling” functions as a gerund and itself is the subject of the embedded sentence. This ambiguity can only be resolved when the listener hears the word that immediately follows the ambiguous phrase, which in this study is always a singular or plural form of the verb “to be,” and which is consistent with one interpretation or the other (e.g., “juggling knives *is*,” or “juggling knives *are*”). Listeners hear the sentence (spoken in a female voice) up to and including the central phrase, and after the offset of the phrase they hear a continuation word (“is”/“are”) spoken in a male voice and indicate whether the word forms a good or bad continuation of the sentence fragment. Note that both continuations are fully grammatical although one is always preferred over the other, as established in pre-tests (see below). Behavioral studies have shown that listeners’ sensitivity to the presence of this type of syntactic ambiguity is reflected in slower responses to the disambiguating word when it follows an ambiguous phrase compared with matched unambiguous phrases (Tyler and Marslen-Wilson, 1977; Tyler et al., 2011).

**Figure 1:**
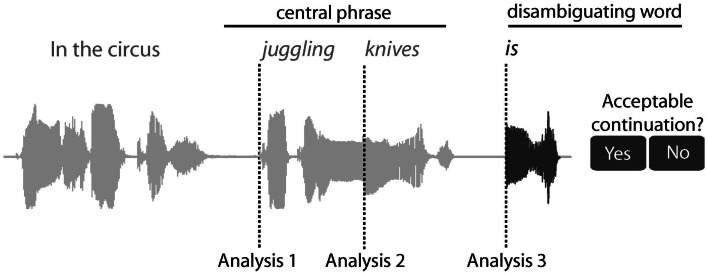
**Sentence structure, task, and different analysis for the RSA analysis**. An example sentence is shown, along with its sound wave, highlighting the central phrase and disambiguating word. After the disambiguating word, participants pressed one of two buttons to indicate whether the disambiguating word was an acceptable continuation for the sentence or not. The RSA analysis was conducted from three positions across the sentence; Analysis 1 from the onset of the central phrase, analysis 2 from the onset of the second word in the phrase, and analysis 3 from the onset of the disambiguating word.

We chart the time-course of the activation and integration of syntactic information by measuring MEG responses at three time-points: from the onset of the central phrase (e.g., “juggling”), the onset of the second word in the phrase (“knives”) and the onset of the disambiguating word (Figure 1). Moreover, by focusing on the time-varying representations within the LH fronto-temporal language system, we can determine how neural computations in the frontal and temporal cortices change over time and investigate their relationship to each other as ambiguity is encountered and resolved.

Implicit in most studies of syntactic ambiguity is the assumption that the activation of lexico-syntactic information, and its integration into the upcoming speech, is involved in processing syntactic ambiguity. Here we directly test these assumptions by using a specific form of multivariate pattern analysis (MVPA), representational similarity analysis (RSA, Kriegeskorte et al., 2008). RSA is founded on analyzing the similarity of brain activation patterns across different items, which serves to characterize the information represented in specific brain regions (Kriegeskorte et al., 2008), but can also uncover how this information changes over time (Su et al., 2012). We construct theoretically motivated models of similarity across the stimuli, based on lexico-semantic, ambiguity activation and ambiguity resolution differences between the stimuli, which we compare against the similarity of activation based on spatio-temporal patterns. This allows us to characterize the types of syntactic computations which occur within the fronto-temporal language network and how they change over time. We focus on two regions of interest (ROIs: LIFG (BA 45/47) and LpMTG) and their RH homologs, functionally defined from a previous fMRI study in which listeners heard a set of stimuli all of which were also included in the present MEG study (Tyler et al., 2011).

## Materials and Methods

### Participants

Thirteen healthy participants took part in the study, with an average age of 23 years (range 19–29 years). All were right-handed with normal hearing. All participants gave informed consent and the study was approved by the Cambridge Psychology Research Ethics Committee.

### Stimuli

The stimuli were 175 spoken sentences, each containing a central phrase of the form “<verb> + ing <noun> + s” (e.g., “juggling knives”; see Table A1 in Appendix). One-third of the sentences contained central phrases that were syntactically unambiguous (e.g., “crying babies”) while the remaining two-thirds consisted of pairs of sentences featuring the two possible readings of syntactically ambiguous phrases (e.g., “In the circus, juggling knives is less dangerous than eating fire” and “In the circus, juggling knives are less sharp than people think”). In all cases, the central phrase was either followed by “is” or “are.” In addition 23 spoken sentences where the central phrase was followed by “was” or “were” were presented as filler items but are not included in the MEG analysis. The sentences were spoken by a female native speaker of British English and recorded in a soundproof booth in a random order, and were then truncated at the end of the central phrase. In a pretest, 23 participants (native British English speakers who did not take part in the main experiment) listened to the sentence fragments and wrote down plausible sentence completions. The proportion of completions consistent with “is” and “are” interpretations was calculated, giving a dominance score for each continuation. For the ambiguous item pairs, one continuation was dominant (i.e., had the higher dominance score) and the other was subordinate, giving 58 dominant and 60 subordinate items. The 57 unambiguous items always had a dominance score of 100% (i.e., the continuation responses were always consistent with the single possible interpretation). The mean [standard deviation (SD)] dominance score was 80% (13%) for the dominant items and 20% (13%) for the subordinate items. The three conditions (subordinate, dominant and unambiguous) were matched on lemma frequency of the two words in the central phrase and on the duration of the sentence fragment.

### Procedure

The participants were seated in a magnetically shielded room (IMEDCO GMBH, Switzerland) positioned under a MEG scanner and fitted with MEG-compatible earphones. Speech was delivered binaurally using ER3A insert earphones (Etymotic Research, Inc., IL, USA) through a pair of semi-flexible plastic tubes fitted with rubber ear inserts. Delays in sound delivery due to tube length and the computer’s sound card were 36 ± 1 ms jitter. This systematic delay was corrected for in the analysis. In the scanner, the sentences were presented in a pseudorandom order with the order of the dominant and subordinate versions of the ambiguous phrases counterbalanced across participants. Each trial consisted of the sentence fragment, followed by a 200 ms silent interval, and then the sentence’s disambiguating word (“is” or “are”) spoken by a male native speaker of British English. One “is” and “are” spoken token was used for all items. Participants were instructed to press a button labeled “yes” with the index finger of their right hand if the disambiguating word was an acceptable continuation of the sentence and a button labeled “no” with the middle finger of their right hand if the disambiguating word was unacceptable. The interval between stimuli was randomized between 1500 and 2500 ms.

Participants were instructed to refrain from blinking or moving their eyes during the presentation of the sentences. To facilitate this, the participants were asked to keep their eyes fixated on a small cross on a back-projected screen positioned 1 m in front of their visual field. The sentences were divided equally into six blocks of 2–3 min duration. Between each block was a short 10–20 s break to allow the participant to blink. The next block was presented when the participant indicated they were ready to continue.

### MEG/MRI recording

Continuous MEG data were recorded using a whole-head 306 channel (102 magnetometers, 204 planar gradiometers). Vector-view system (Elekta Neuromag, Helsinki, Finland) located at the MRC Cognition and Brain Sciences Unit, Cambridge, UK. Eye movements and blinks were monitored with electro-oculogram (EOG) electrodes placed around the eyes, and five Head Position Indicator (HPI) coils were used to record the head position (every 200 ms) within the MEG helmet. Electro-cardiogram (ECG) electrodes were placed on the right shoulder blade and left torso to record cardiac muscular effects. The participants’ head shape was digitally recorded using a 3D digitizer (Fastrak Polhemus Inc., Colchester, VA, USA) using 70–100 points, along with the positions of the EOG electrodes, HPI coils, and fiducial points (nasion, left and right periauricular). MEG signals were recorded at a sampling rate of 2000 Hz and between 0.01 and 667 Hz. To facilitate source reconstruction, 1 mm × 1 mm × 1 mm T1-weighted MPRAGE scans were acquired during a separate session with a Siemens 3T Tim Trio scanner (Siemens Medical Solutions, Camberley, UK) located at the MRC Cognition and Brain Sciences Unit, Cambridge, UK.

### MEG processing

Initial processing of the raw data used MaxFilter version 2.2 (Elektra-Neuromag) to detect static bad channels that were subsequently reconstructed along with any bad channels noted during acquisition or from visual inspection of the raw data afterward (between 4 and 15 bad channels). The temporal extension of the signal-space separation technique (SSS; Taulu et al., 2005) was applied to the data every 4 s in order to segregate the signals originating from within the participants’ head from those generated by external sources of noise. Head movement compensation (using data from the HPI coils) was performed, and the head position was transformed into a common head position to facilitate group sensor analyses.

The remaining processing used SPM8 (Wellcome Institute of Imaging Neuroscience, London, UK). The MEG data were down-sampled to 500 Hz and low pass filtered at 40 Hz using a bi-directional 5th order Butterworth digital filter. The continuous data were then divided into epochs at each of the three trigger points (Figure 1): first, from the onset of the central phrase (from −100 to 1000 ms as the mean length of the central phrase is 1070 ms); second, the onset of the second word in the central phrase (from −100 to 500 ms as the mean length of the second word is 566 ms); and third, the onset of the disambiguating word (from −100 to 800 ms, based on the latencies of behavioral responses). The baseline was defined as the average response between −100 and 0 ms relative to stimulus onset. The average response from the baseline period (−100 to 0 ms) was subtracted from all data points in the epoch. Using a baseline immediately prior to each epoch should help normalize effects accumulating before the onset of each word, so that each analysis is optimized to identify effects brought about by the epoch (or the offset of the previous word).

Automated artifact detection and visual inspection was used to exclude bad epochs. Epochs were excluded if the data were flat (zeroes) or if unusual steps were detected. With the remaining epochs, independent components analysis was used to remove artifactual signals generated by the eye movements or cardiac signals present in the MEG data by removing components that showed significant correlations with the vertical and horizontal EOG and ECG electrodes. A bootstrap permutation approach was used to determine the significance of the correlations.

### Source reconstruction

The data were prepared for MEG ROI analysis (see ROI definitions) by constructing a source model over the cortical mesh surface for each participant. Structural MRI images were segmented and transformed to an MNI template brain using SPM8. Using the inverse transformation, individual scalp and cortical meshes were then constructed by warping canonical meshes of the MNI template to the participant’s MRI space. Co-registration between the MEG sensor coordinates and the participant’s MRI coordinates was achieved by aligning the digitized head and fiducial points to the outer scalp mesh. Source reconstruction used a cortically constrained minimum norm model in SPM8 with a single shell conductor model. The inversion was computed over the whole epoch and all models accounted for more than 95% of variance. From the resulting source models, the moment at any mesh point (vertex) may be extracted as a time-course over the epoch. We extracted the time-course of each vertex within each of our ROIs (see ROI definitions) that were then used for the RSA analysis.

### RSA analysis

Representational similarity analysis involves testing models of the information content of the stimuli by comparing the dissimilarity structure of the stimuli predicted by those models to the dissimilarity structure present in neural activation patterns. We constructed a number of representational dissimilarity matrices (RDMs), sensitive to the different kinds of information that we hypothesize is important at different points in the activation and resolution of syntactic ambiguity.

**Figure 2:**
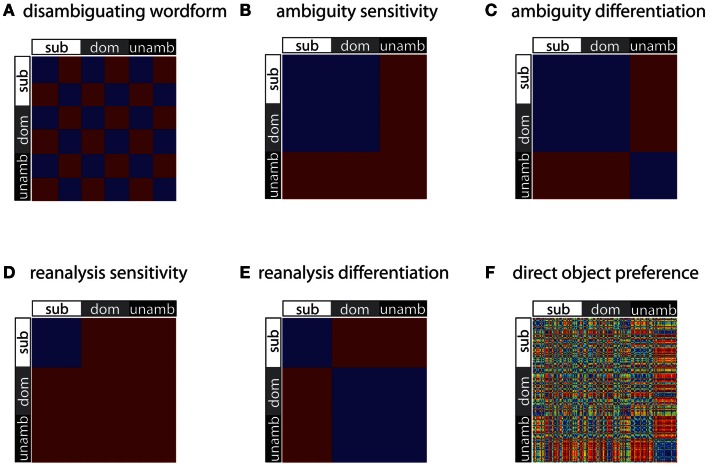
**Model RDMs used in the analyses**. Each RDM is a 198 × 198 matrix, with each entry being either a 0 (meaning no dissimilarity, depicted as blue) or 1 (meaning maximal dissimilarity, depicted as red). These RDMs test for differences in representational similarity across stimuli – for wordform **(A)**, syntactic ambiguity **(B, C)**, and syntactic reanalysis **(D, E)**. Note that direct object preference **(F)** is a continuous-valued measure, and so dissimilarities based on object preference take on a range of values, from 0 to 1.

The first of these models (“*disambiguating wordform*”) is sensitive only to the identity of the disambiguating word; two stimuli are modeled as similar if and only if the same disambiguating wordform (“is” or “are”) was used with them. As disambiguating word identity was counterbalanced over the experimental conditions, this RDM is orthogonal to the three conditions of subordinate, dominant, and unambiguous (Figure 2A). This model distinguishes the acoustically different disambiguation words and was primarily included as a check on the sensitivity of the analysis method, since this RDM should correlate with similarity of activation patterns in auditory cortex. Such acoustic models were not generated for the two central phrase words, because each trial has unique acoustic information during these epochs.

The other RDMs presented in Figure 2 test for differences due to syntactic processing, such as effects due to competition between parse possibilities and effects due to syntactic reanalysis when the disambiguating verb is inconsistent with the preferred interpretation of the central phrase. The *ambiguity sensitivity* RDM (Figure 2B) tests whether ambiguous items, irrespective of the dominance of the subsequent disambiguating word, give rise to similar activation patterns. Common to the ambiguous items is that they are associated with multiple possible syntactic analyses and the potential competition between them, and this model assumes that this processing results in a specific pattern of neural activity for the ambiguous items. Since the unambiguous items are not associated with multiple meanings, the neural patterns for these items are hypothesized to be uncorrelated, and so are modeled as dissimilar in this RDM.

**Figure 3:**
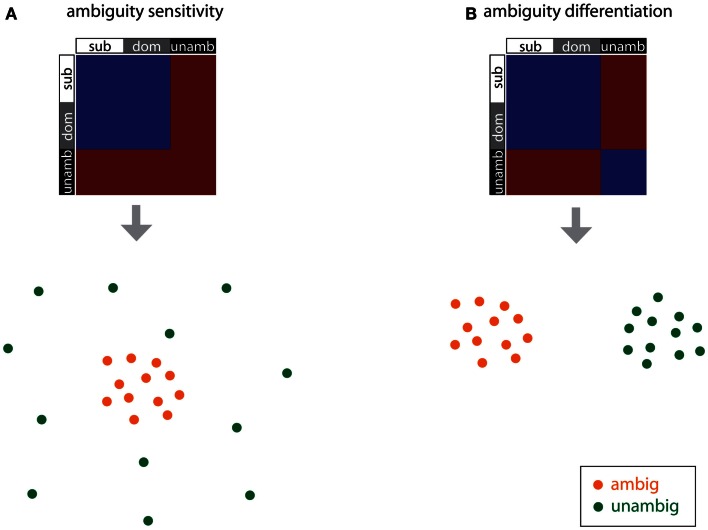
**RDMs and corresponding cartoon multidimensional scaling plots for the ambiguity sensitivity and ambiguity differentiation models**. **(A)** The *ambiguity sensitivity RDM* tests the hypothesis that a distinct pattern of neural activity, associated with the processing of multiple syntactic parses, arises for the ambiguous items (i.e., the subordinate and dominant conditions). This pattern of neural activity does not arise for unambiguous items and so unambiguous items have uncorrelated activation patterns (dissimilar to each other and also dissimilar to the ambiguous items). **(B)** The *ambiguity differentiation RDM* tests the hypothesis that ambiguous items and unambiguous items give rise to different distinct patterns of activation: ambiguous items are similar to each other, unambiguous items are similar to each other, and ambiguous and unambiguous items differ.

The *ambiguity differentiation* RDM is the same as the ambiguity sensitivity RDM, except that pairs of unambiguous items are also modeled as similar to each other (Figure 2C). Note that the *ambiguity differentiation* RDM tests for differences between the activation patterns for ambiguous and unambiguous items, whereas the *ambiguity sensitivity* RDM test for specific patterns of activation associated with the processing of ambiguity (Figure 3). Furthermore, presence or absence of ambiguity is a property of the central phrase itself, independent of the identity of the subsequent disambiguating word, and for this reason subordinate and dominant items are modeled in the same way for this pair of RDMs.

The next pair of models test for differences due to syntactic reanalysis. The *reanalysis sensitivity RDM* tests whether the subordinate items (for which the central phrase is followed by a disambiguating verb consistent with the less dominant interpretation), give rise to similar patterns of activation (Figure 2D). For the subordinate items, the competition between multiple possible syntactic readings is resolved in favor of the less preferred reading, and so these items require a process of revision or reanalysis in order to correctly integrate the disambiguating wordform with the preceding sentence fragment. This model assumes that this process of reanalysis and integration gives rise to a specific pattern of neural activation for these items. The *reanalysis differentiation RDM* is the same as the *reanalysis sensitivity* RDM, except that items which do not require reanalysis (i.e., dominant and unambiguous) are modeled as being similar to each other; this model thus differentiates items requiring reanalysis from those which do not (Figure 2E).

According to lexicalist accounts of sentence processing, lexico-syntactic knowledge associated with each word guides activation of candidate parses and should therefore be influential in both the creation of local ambiguities and in the ambiguity resolution process (Tyler and Marslen-Wilson, 1977; Marslen-Wilson et al., 1988; MacDonald et al., 1994). For example, verb subcategorization frame (SCF) preferences may affect sentence processing by placing constraints on how potential arguments are incorporated into the emerging representation. Furthermore, it has often been hypothesized that such knowledge reflects statistical data on words’ usage in language (Merlo, 1994; Garnsey et al., 1997; Lapata et al., 2001). The final RDM we included in our analyses was designed to be sensitive to lexico-syntactic properties of the verb used in the first word of the central phrase (e.g., “juggle” in “juggling knives”) because we hypothesized that verbs with different lexico-syntactic properties would give rise to different patterns of activation. In particular, we hypothesized that verb subcategorization behavior would be one factor influencing processing as the central phrase is being heard. For verbs with a high probability of occurrence with noun-phrase direct object complements (e.g., “mark”) we predicted a preference to interpret the first word of the central phrase as a gerund, because in such cases the following noun is likely to function as the verb’s theme (e.g., “marking essays”), whereas for verbs with a low probability of occurrence with direct object complements there would be a stronger preference for adjectival readings (e.g., “yawning audiences”). Given these considerations, we predicted that verbs with different likelihoods of taking direct object complements should show different patterns of activation.

**Figure 4:**
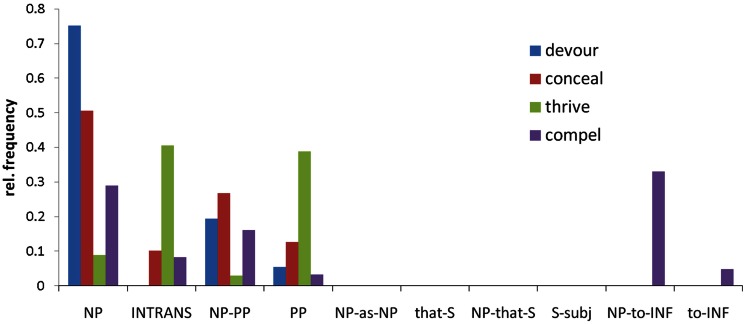
**The relative frequency distributions for four example verbs over the 10 most common subcategorization frames, which are labeled by a description of their argument structure**. Different verbs have different distributions, illustrating the differences in their subcategorization frame behavior. NP, noun-phrase complement (e.g., “he devoured the meal”); INTRANS, intransitive (e.g., “he thrived”); PP, prepositional phrase complement (e.g., “he thrived in school”); S, sentential complement; INF, verb infinitive.

To obtain SCF frequency distributions for each verb we used VALEX, an automatically acquired subcategorization lexicon for 6,397 English verbs that is derived from large, cross domain corpora (Korhonen et al., 2006). Earlier studies have typically estimated lexico-syntactic information using behavioral pre-tests; however, the extent to which such approaches truly reflect statistical information in the language is unclear. VALEX includes statistical information about the relative frequency of occurrence of each of 163 possible SCF types with each verb (Figure 4). For each verb and SCF pair, the lexicon also gives the syntax of the arguments (for example, subject or complement), as well as the part-of-speech tags and lexical tokens found for those arguments for all instances of the verb in the corpus.

The 163 SCFs were partitioned into those that specify NP direct object complements and those which do not, and the total relative frequency of frames specifying NP direct object complements for each verb was calculated (Table 1). Given our prediction that verbs with a high probability of occurrence with direct object complements would show different patterns to those with low probability of occurrence with direct object complements, we calculated dissimilarity between pairs of stimuli as the absolute difference in their direct object probability scores (Figure 2F). Note that this RDM only incorporates information about the verb’s lexico-syntactic behavior; in particular it does not contain information about the noun that follows it, nor does it contain information about the subsequent disambiguating word.

**Table 1:** **Direct object preference scores (“DO pref”), calculated from VALEX, for each of the verbs used in the study**.

Verb	DO pref	Verb	DO pref	Verb	DO pref	Verb	DO pref
Cling	0.00	Fly	0.27	Neglect	0.54	Install	0.80
Ache	0.00	Flash	0.29	Accelerate	0.54	Bring	0.80
Work	0.01	Fail	0.30	Spin	0.55	Juggle	0.80
Function	0.05	Shrug	0.31	Clean	0.56	Torture	0.81
Rhyme	0.08	Crash	0.32	Prevent	0.56	Conquer	0.83
Differ	0.08	Capsize	0.33	Wake	0.57	Establish	0.83
Chuckle	0.10	Camp	0.34	Breed	0.58	Bully	0.84
Laugh	0.10	Advance	0.36	Roast	0.58	Insult	0.85
Yawn	0.10	Pass	0.36	Train	0.61	Blame	0.85
Sneer	0.10	Sail	0.36	Pickle	0.65	Salute	0.86
Struggle	0.10	Cheat	0.36	Read	0.66	Select	0.87
Interfere	0.11	Manage	0.37	Cut	0.66	Charm	0.87
Emerge	0.11	Predict	0.37	Reverse	0.67	Inspire	0.88
Quarrel	0.12	Hunt	0.38	Worry	0.70	Mock	0.88
Glisten	0.16	Speed	0.39	Bandage	0.70	Appoint	0.89
Glow	0.16	Advertise	0.41	Kick	0.70	Interest	0.89
Despair	0.16	Regret	0.42	Pack	0.71	Irritate	0.90
Cry	0.16	Drown	0.42	Allow	0.72	Adopt	0.90
Grow	0.17	Discern	0.45	Inherit	0.72	Exploit	0.91
Joke	0.17	Impress	0.46	Rent	0.72	Provoke	0.92
Ripen	0.18	Sink	0.49	Chase	0.72	Resolve	0.92
Rise	0.19	Slam	0.49	Park	0.73	Mark	0.93
Live	0.20	Clink	0.49	Build	0.74	Bribe	0.94
Explode	0.23	Play	0.50	Release	0.77	Stimulate	0.95
Land	0.24	Trust	0.50	Attack	0.77	Denounce	0.95
Travel	0.24	Boil	0.51	Imprison	0.77	Harm	0.96
Walk	0.25	Rehearse	0.52	Employ	0.77	Disturb	0.96
March	0.26	Cook	0.52	Acquire	0.77		
Flower	0.27	Describe	0.52	Shred	0.77		

### ROI definitions

**Figure 5:**
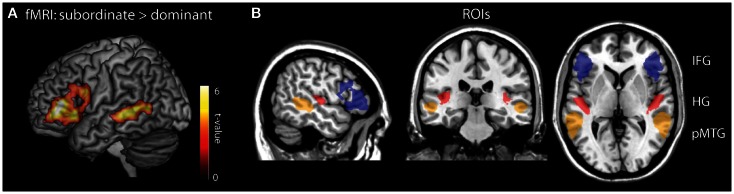
**Regions of interest (ROIs) used in the MEG RSA analysis**. **(A)** Functional ROIs were obtained from an fMRI contrast of subordinate > dominant sentences. **(B)** The entire complement of ROIs used included the fMRI defined LIFG (blue) and LpMTG (orange) with the anatomically defined Heschl’s gyrus (red). Each region also has a right hemisphere homolog.

Our ROIs for the MEG analysis were functionally defined from a previous fMRI study in which 15 independent participants (aged 19–24 years) heard the stimuli included in the MEG study. Unlike in the present study, the participants in the fMRI study merely attentively listened to the sentences instead of performing a task, and they heard the entire sentence without disruption (see Tyler et al., 2011 for scanning details). Consistent with previous research, fMRI analysis showed increased activity in left BA45/47 and left posterior MTG during subordinate compared to dominant sentences (voxelwise *p* < 0.01, cluster *p* < 0.05; Figure 5A), In order to test for potential bilateral contributions to syntactic analysis we also created right hemisphere homologs of these LH ROIs Figure 5B. To provide a baseline for testing the efficacy of the ROI RDM approach, we also included anatomically defined ROIs of bilateral Heschl’s gyrus (HG) which we predicted would show sensitivity effects for the disambiguating wordform RDM.

### Neurocognitive predictions

In the RSA, the goal is to chart the time-course of the different kinds of processing involved in local ambiguity resolution by testing for effects of these RDMs during the three different epochs. The onsets of these three epochs are defined with respect to three key trigger points within the stimuli where different kinds of linguistic information are available. For the earliest epoch (the first word in the central phrase), we predict effects associated with the activation of verb lexico-syntactic knowledge, but only after the lexical identity of the word has been established (e.g., after the word’s recognition point; Marslen-Wilson, 1987) or during the processing of the second word in the central phrase. Given the lexical nature of the direct object preference measure, and on the assumption that posterior middle temporal regions represent lexical-level information relevant to processing verbs in context (Hickok and Poeppel, 2007; Tyler et al., 2008; Rodd et al., 2010), these direct object preference effects are most likely to be seen in LpMTG.

In contrast, the ambiguity RDMs should not show effects during processing of the first word in the central phrase, because the ambiguity RDMs are sensitive to the ambiguity of the phrase, not the ambiguity of the first word. In fact, during the first epoch, both adjectival and gerundive usages are still possible for all stimuli, including those in the unambiguous condition. For example, “crying babies” is one of our unambiguous phrases, requiring an adjectival reading, but, at the point “crying” is heard, both adjectival and gerundive continuations are still possible (for example, the sentence may continue “crying loudly is …”). The distinctions captured by the ambiguity differentiation and ambiguity sensitivity RDMs do not exist during the first epoch. This is not to say that there are no differences between items at this point that may affect processing, but such differences must necessarily be properties of the first word alone. The direct object preference measure is one example of such a property.

During the second epoch, information about the second word in the central phrase becomes available. Once the second word has been recognized, the stimuli are distinguished by whether or not the central phrase is ambiguous, and so the detection of an ambiguity, or the concurrent access of multiple representations associated with the ambiguity, should disassociate neural activation patterns for the ambiguous items (subordinate and dominant conditions) from the activation patterns for the unambiguous items. We therefore predict effects of the two ambiguity RDMs during the second epoch. As mentioned above, we also predict continued lexical effects associated with the direct object preference RDM, as the preceding verb’s probability of taking a direct object influences the likelihood of incorporating the noun as the verb’s theme or agent. Furthermore, as the activation of different possibilities for the verbs SCF behavior are a key factor causing local ambiguity, we hypothesize that effects for the direct object preference RDM should precede effects associated with the ambiguity models.

The first two epochs were designed so that they did not overlap with the onset of the disambiguating word, and so for these epochs there is no information about how the ambiguity is ultimately resolved. We therefore do not test RDMs that are sensitive to the identity of the disambiguating wordform in the analyses for the first two epochs (i.e., we excluded the disambiguating wordform RDM and the two reanalysis RDMs).

The third epoch begins at the onset of the disambiguating word, and is designed to include the resolution of the local ambiguity. As the disambiguating words were either “is” or “are,” the disambiguating wordform RDM represents whether these words are acoustically the same or different, and we thus predict that this RDM will correlate most strongly with activation patterns in auditory cortex at early time-points in the third epoch. We also predict early effects of ambiguity during this epoch, as competition between the multiple candidate representations that arise from ambiguous central phrases is processed in inferior frontal cortex. We anticipate that later processes of reanalysis and integration will depend on LIFG (Hagoort, 2005). Whether the LpMTG is also involved in ambiguity resolution and reanalysis remains to be determined. Although the LpMTG co-activates with the LIFG in fMRI studies of syntactic ambiguity, fMRI does not enable the various processes involved in activation, selection, and reanalysis to be separated out and therefore there are no clear predictions from previous studies concerning the role of the LpMTG in ambiguity resolution.

### MEG ROI representational dissimilarity matrices

These six theoretically motivated RDMs were statistically compared to RDMs derived from the source localized ROI data. For each ROI we extracted the time-course of each vertex for each trial that was used to construct the MEG-based RDMs.

Here we used a sliding time-window approach, where for one time-point, the MEG data for all vertices and all time-points ±50 ms are concatenated into a single vector (length = vertices × time-points). We then calculated the dissimilarity between all item pairs using 1 – Pearson’s correlation as a distance measure. Therefore, each MEG-based RDM incorporates data from all vertices without averaging across them and reflects dissimilarity based on spatio-temporal patterns. This process was then repeated for all time-points resulting in one RDM per time-point for each ROI. The MEG-based RDMs were then correlated with the relevant theoretical model RDMs using Spearman’s rank correlation to obtain a similarity time-course reflecting the relatedness of the two dissimilarity matrices. A single time-course was obtained per model RDM per participant, at each of the three trigger points. To evaluate whether each model RDM was significantly reflected in the MEG data across the group, a one-sample *t*-test was conducted at each point in time (alpha = 0.05), and corrected for multiple comparisons using cluster-based permutation testing (Maris and Oostenveld, 2007). We only report effects which are cluster-level significant at 0.05 unless noted.

## Results

### Behavioral data

We analyzed participants’ rejection rates – i.e., the frequency with which they rejected the disambiguating word as an acceptable continuation of the sentence fragment. The rejection rates were analyzed using a repeated measures ANOVA with three conditions (subordinate, dominant, and unambiguous). There was a main effect of condition [*F*(2, 24) = 24.17; *p* < 0.001], with the largest proportion of unacceptable decisions for the subordinate (34%), fewer for dominant (13%), and the least for the unambiguous condition (6%). The RTs showed a similar pattern, with a main effect of condition [*F*(2, 24) = 8.72; *p* = 001] and judgment latencies to the subordinate continuations (863 ms) being longer than for the dominant continuations (820 ms) which in turn were longer than the unambiguous sentences (769 ms). These results suggest that participants initially base their analysis on the preferred interpretation of the ambiguous phrase (the dominant reading) which then has to be revised when they encounter a disambiguating word which is inconsistent with that interpretation. This requirement to reinterpret leads to many items being judged as unacceptable and slower decision latencies. Performance in this task provides a measure of participants’ sensitivity to syntactic information during the processing of a spoken sentence (Tyler et al., 2011).

### MEG analyses

In order to test the time-course of activation and integration of syntactic information in the frontal-temporal language network, we performed an RSA analysis on the MEG data at three positions within the spoken sentence (Figure 1). By comparing the similarity of MEG activity patterns to those predicted by different properties of the sentence, we can uncover the kinds of processes different regions are engaged in and how they evolve over time.

### Effects during the central phrase

Our initial RSA analyses aimed to determine the kinds of information processed within the fronto-temporal language network while participants listened to the central phrase section of the sentence. Our first analysis tested for effects of the activation of lexico-syntactic knowledge linked to the first verb in the central phrase, and for ambiguity effects that may arise during the central phrase, however our RSA analysis failed to find significant effects for any of these model RDMs. This may be because the ambiguity in the phrase is more closely linked to the second word of the phrase, at which point the central phrase becomes ambiguous (e.g., when *planes* is heard in the phrase *landing planes*) or unambiguous (e.g., when *babies* is heard in the phrase *crying babies*). Effects relating to the activation of lexico-syntactic knowledge cannot be activated immediately upon the onset of the first word, but become available gradually over time as sufficient acoustic information accrues and the word can be recognized (Marslen-Wilson, 1987). Therefore, they may not be detectable until later in the word.

**Figure 6:**
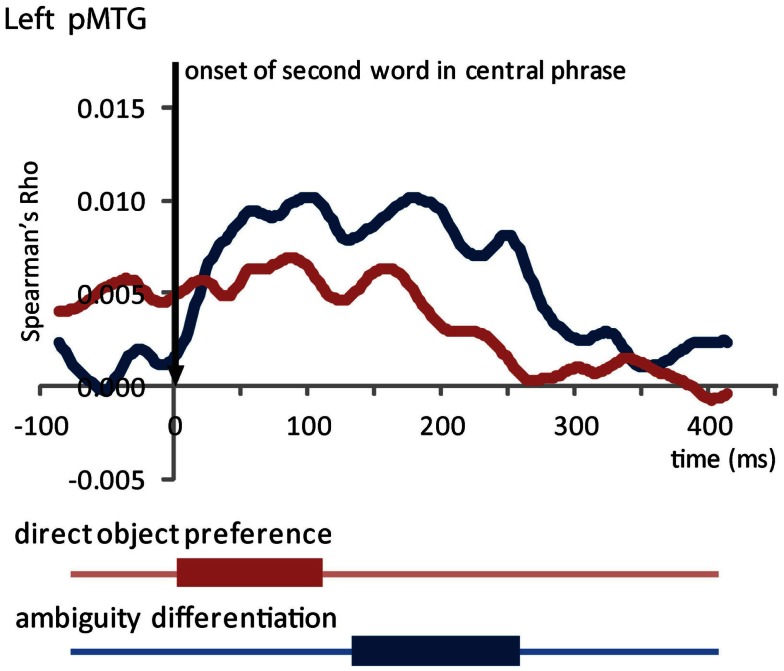
**Effects in the LpMTG from the onset of the second word in the central phrase**. Plot shows the time-course of similarity between the model RDM and the LpMTG RDMs. Time periods of significant similarity are shown below plot by solid bars.

From the onset of the second word in the central phrase, our RSA analysis revealed two marginally significant effects in the LpMTG (Table 2; Figure 6). We found a similarity effect in the LpMTG that matched the direct object preference RDM from the onset of the second word to 118 ms post-onset. The rapid nature of this effect means it is likely to be a reflection of similarity patterns representing the activation of the first word’s lexico-syntactic properties. Although these effects are only marginally significant we report them here and include them in our on interpretation of the results because we believe them to be reliable and interpretable in relation to current models of language processing.

**Table 2:** **RSA results from the onset of the second word in the central phrase**.

Model	ROI	Start	Stop	cluster p
Direct object preference	LpMTG	2	118	0.064
Ambiguity differentiation	LpMTG	136	264	0.059

*Start and end times of effects are shown and reflect the central time-point of the 100 ms sliding time-window*.

In addition, we also found an effect in the LpMTG of the ambiguity differentiation RDM from 136 to 264 ms after the onset of the second word. There were no effects in the LIFG (no clusters identified) or the RH (RIFG *p*’s > 0.15, RpMTG no clusters identified, RHG *p*’s > 0.14) and no further effects in LpMTG (all *p*’s > 0.2). This analysis shows that during the central phrase, the LpMTG activation patterns shift from representing the lexico-syntactic information about the first word in the central phrase to reflecting the degree of ambiguity in the central phrase as more of the phrase is heard. The accumulated ambiguity contained in the central phrase can only be resolved once the subsequent disambiguating word form is heard.

### Effects at the point of disambiguation

In order to determine the kinds of information processed when participants hear the disambiguating word which initiates the resolution of the preceding ambiguity, we performed RSA time-course analysis from the onset of the disambiguating word by testing model RDMs capturing ambiguity and reanalysis (Table 3).

**Table 3:** **RSA results from the onset of the disambiguating word**.

Model	ROI	Start	Stop	Cluster *p*
Disambiguating wordform	LHG	30	148	0.049
	LHG	236	396	0.004
	RHG	58	250	0.026
	RHG	396	518	0.052
Disambiguating wordform	LpMTG	44	198	0.026
	LpMTG	216	392	0.025
	LpMTG	492	624	0.049
	RpMTG	430	584	0.042
Ambiguity sensitivity	LIFG	36	190	0.054
Ambiguity differentiation	LIFG	302	708	0.012
Reanalysis sensitivity	LIFG	374	714	0.014

*Start and end times of effects are shown and reflect the central time-point of the 100 ms sliding time-window*.

**Figure 7:**
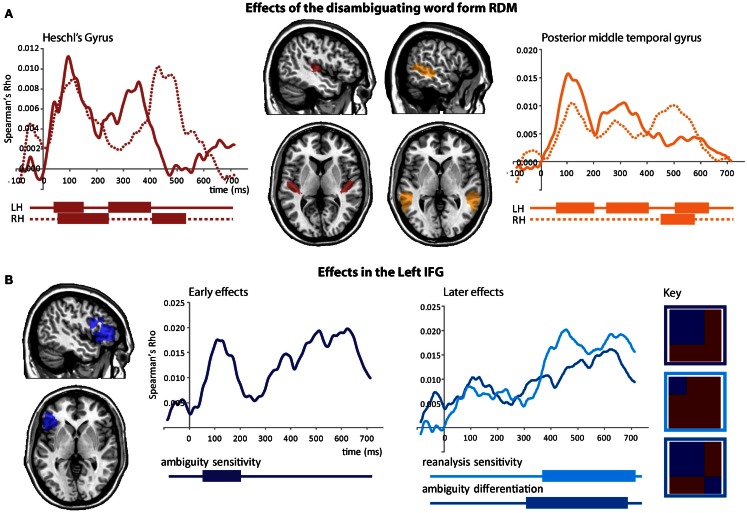
**Effects from the onset of the disambiguating word**. **(A)** Effects of the disambiguating word form RDM captures similarity according to the acoustic input which is found in bilateral HG and pMTG. Plots show time-course of similarity between the disambiguating word form RDM and HG RDMs (red), and pMTG RDMs (orange). Significant effects are shown below plots by solid bars. **(B)** Early and later effects in the left IFG for ambiguity RDMS and the syntactic reanalysis RDM.

We first tested whether activity patterns in primary auditory cortex follow the similarity structure defined by the acoustic input (i.e., the disambiguating wordform). We found early similarity effects in bilateral HG that reflected the similarity structure predicted by the disambiguating wordform (i.e., “is” or “are”; Figure 7A, left), showing that responses in primary auditory regions reflect the auditory input, peaking around 94 ms, and showing a recurring pattern over time. A similar early effect was seen in LpMTG peaking at 106 ms. Subsequent peaks in the RH occurred approximately 200 ms later than those in the LH (Figure 7A, right). There were no further effects in either HG or pMTG and no further effects for the disambiguating wordform model [LHG *p*’s > 0.26, RHG *p*’s > 0.33, LpMTG *p*’s > 0.45, RpMTG *p*’s > 0.19, wordform model *p*’s > 0.13 (LIFG)].

**Figure 8:**
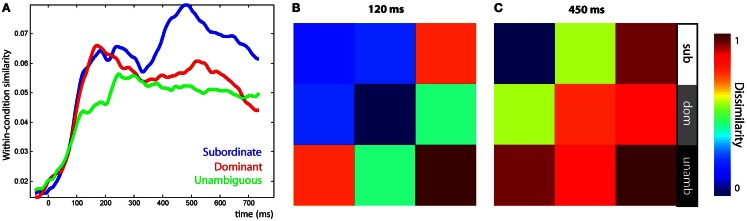
**Visualization of similarity patterns in the LIFG from the onset of the disambiguating word**. **(A)** Within-condition similarity time-courses show the group average similarity between items from the same conditions, plotted over time. **(B)** RDM of the LIFG after 120 ms shows ambiguous items are self-similar before, **(C)** subordinate items become self-similar, shown at 450 ms. RDMs show the average similarity within each of the nine conditional combinations.

Three RSA effects were found in the LIFG, each relating to various aspects of syntactic ambiguity. There was an early significant effect, peaking around 120 ms, for the ambiguity sensitivity RDM (Figure 7B, left). This finding was confirmed by visualizing the LIFG similarity patterns at 120 ms which showed that all ambiguous items were more similar to other ambiguous items than to unambiguous items, with no differentiation between subordinate and dominant continuations (Figure 8B). This was also evident by tracking the within-condition similarity over time, where early time periods showed subordinate and dominant items have a similar degree of within-condition similarity that was greater than the within-condition similarity for the unambiguous items (Figure 8A). These results show the early activation in the LIFG represents the processing of ambiguity associated with both the subordinate and dominant sentence continuations.

There were two significant late effects in the LIFG occurring between 300 and 700 ms associated with the ambiguity differentiation and reanalysis sensitivity RDMs (Figure 7B, right). Ranking the two models by their fit to the MEG data showed the top model was the reanalysis sensitivity RDM that captures high similarity within the subordinate items and low similarity within both the dominant and unambiguous items. This sensitivity to subordinate items is confirmed by visualizing the data RDM after 450 ms that shows a subordinate sensitive pattern (Figure 8C). Further, the within-condition similarity time-course shows the subordinate items are more similar to each other than either the dominant or unambiguous items, a pattern that emerges after 300 ms (Figure 8A). These results suggest that as listeners integrate the sentence fragment with the disambiguating word, the LIFG is initially sensitive to the presence of multiple representations carried by the phrase that were previously represented in the LpMTG. The activation of multiple representations when the disambiguating word is heard may trigger competitive activation in the LIFG. Only later, as the ambiguity begins to be resolved, is the LIFG sensitive to the difference between subordinate and dominant readings, reflecting their different integration demands.

In summary, the RSA analysis from the onset of the disambiguating verb shows information relating to the verb-form in HG and pMTG within 100 ms. Subsequently, peaking at 120 ms the LIFG represents the ambiguous items (both subordinate and dominant items) in a similar fashion, and differentiates them from the unambiguous items. The posterior MTG and HG then show evidence for the reactivation of representations of the disambiguating word, before finally the LIFG shows sensitivity to representing information about subordinate items that require additional reanalysis.

## Discussion

Although previous studies have established the importance of functional connectivity between LIFG and LpMTG in syntactic processing, the differential roles that these two regions play, the types of computations they subserve and the functional relationship between them remain unclear. To address these issues, in the present study, we constructed sentences containing syntactically ambiguous and unambiguous phrases and tested a variety of RDMs reflecting lexico-syntactic information, ambiguity sensitivity and ambiguity resolution against the similarity of neural spatio-temporal activation patterns using MEG. The results show that the LpMTG is sensitive to both the form of a word and its lexical properties. The LIFG, in contrast, appears blind to these features of the speech input and instead responds to the competitive consequences of multiple syntactic representations and determines their resolution. These processes appear to be largely sequential with information flowing from LpMTG to the LIFG.

**Figure 9:**
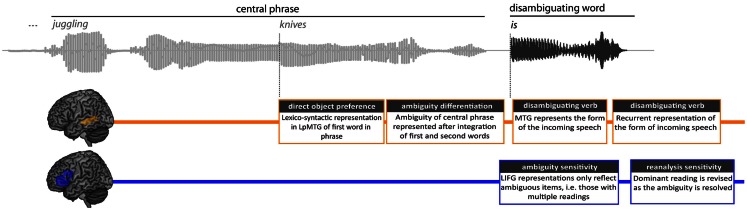
**Summary of results in the left fronto-temporal language network showing RSA effects in the LpMTG and LIFG during the central phrase and the subsequent disambiguation**.

This pattern of results is revealed in the multivariate similarity structure at each epoch (see Figure 9 for summary). We found no effects of any of the model RDMs at the first word in the central phrase, but marginal effects of both lexico-syntactic properties and ambiguity were seen during the second word, suggesting that the ambiguity status of the central phrase is only determined when both words in the phrase have become available. Although these effects were only marginal, we believe them to be relevant and interpretable in the context of current models of on-line language processing. One factor that may contribute to the weakness of some effects is in the inherent difficulty of obtaining accurate word-onsets from the continuous speech signals. Given the sensitivity of MEG to variations in the acoustic signal, even small discrepancies can influence the results. As a result, the majority of studies employing sentence paradigms use written text presented one word at a time, however this processes is undeniably very different to naturalistic language comprehension. Here we analyze points within continuous speech to alleviate this problem though other issues such as reduced signal-to-noise and word onset variability may count against us.

The effect of the lexico-syntactic RDM, located in the LpMTG and first seen toward the onset of the second word, reflects the lexico-syntactic properties of the preceding word which are captured in the VALEX-derived estimates of the frequency with which a verb takes a direct object (the direct object preference RDM). The earliness of these lexico-syntactic effects suggest that the LpMTG may be sensitive to the integration of the properties of the two words in the phrase, with the first word’s lexically based syntactic constraints being reflected in the early processing of the following noun. These results are consistent with lexically driven models of sentence processing which claim that as each word is heard the properties associated with that word start to be activated and integrated into the upcoming sentence (Marslen-Wilson, 1973; Marslen-Wilson and Tyler, 1980; MacDonald et al., 1994). At this point in time, no such effects were seen in the LIFG. Only the LpMTG appeared to be sensitive to lexically driven information, a finding consistent with claims that lexical representations are associated with the LpMTG (Indefrey and Cutler, 2004; Hagoort, 2005; Thompson et al., 2007; Tyler et al., 2008; Snijders et al., 2010).

It is only after the lexical properties of the first and second words have been integrated that the phrases are distinguished by whether or not they are ambiguous. Consistent with this, slightly later in the processing of the second word of the phrase, around 136 ms, we start to see the patterns of activation in the LpMTG shift from reflecting the lexico-syntactic properties of the first word to the properties of the ambiguity differentiation RDM. At this point in time, the phrase’s ambiguity becomes established. The ambiguity differentiation RDM discriminates between the ambiguous and unambiguous items, while treating the subordinate and dominant items the same, suggesting that the LpMTG is responsive to the commonality between the two sets of ambiguous items – namely, that they are both associated with multiple syntactic interpretations. One possibility suggested by this pattern of results is that effects in the LpMTG may not require the involvement of the LIFG. Changing sensitivity to different aspects of the input over time may not be under the dynamic control of the LIFG, and sustained activation of the LpMTG may not always be modulated by the involvement of the LIFG during integration (see also Snijders et al., 2010).

The LIFG only showed sensitivity to RDMs when the disambiguating wordform was heard. This is the earliest point at which the ambiguity can be resolved and it is here that the LIFG seems to play a major role. Early in the processing of the disambiguating word, there are recurrent effects of the phonological form of the verb in bilateral HG between 30 and 400 ms with slightly later effects in the LpMTG. Although the verb effects in LpMTG peaked slightly later than HG, suggesting a flow of information, it could also be that both ROI effects originate from the same the underlying source. To fully address this issue would require evidence from a more spatially accurate approach (e.g., fMRI). Although the LIFG is not sensitive to these form-based processes, it does show early effects of the ambiguity sensitivity RDM followed by the ambiguity differentiation RDM, perhaps in response to earlier ambiguity effects in the LpMTG during the second word in the phrase. The LIFG’s sensitivity to the ambiguity is soon followed by its resolution, where the LIFG is critically involved in the reanalysis required when the disambiguating verb is consistent with the less dominant interpretation.

We found no evidence that the LIFG showed any interest in the activation and integration of lexico-syntactic information within the central phrase, or in the effects of ambiguity which became available once the words in the central phrase were integrated, during the processing of the second word in the central phrase. The LIFG only became involved in the processing of ambiguity resolution when triggered by the presence of the disambiguating word. Moreover, the role of the LIFG seems to be quite specific; it only became involved in the integration of upcoming words when the disambiguating word occurred, requiring the current (dominant) interpretation of the sentence to be revised. This suggests in turn that the LIFG is involved in detecting the presence of a structural ambiguity that requires resolution and/or selection between the syntactic possibilities in the context of the disambiguating word. One interpretation of these results is that they argue against those models which assume that the LIFG inevitably operates in a top-down fashion to guide interpretation (Federmeier, 2007), or to maintain or update representations in the LpMTG. It looks from these results as though the LIFG is primarily responsive to processes involving competition and re-evaluation, and that it might not always be involved in processes of integration, when lexical representations need to be combined to form a structured sequence. The present results give no evidence for the LIFG supporting the integration between the words in the central phase, as the LIFG showed no sensitivity to the ambiguity or to lexical integration processes during the processing of the second word in the phrase. Further studies are required to fully establish the role of the LIFG in on-line language processing.

The results of this study go some way to addressing an important issue left unanswered by previous fMRI studies of fronto-temporal connectivity during syntactic processing (Snijders et al., 2010; Papoutsi et al., 2011), concerning the dynamic interplay between LpMTG and LIFG. While the analyses reported here suggest that information flows one way from LpMTG to LIFG, they are not unequivocal. However, further support for this claim comes from an independent set of analyses on the MEG data in which we carried out time-frequency analysis and phase locking analyses and then computed Granger causality measures to determine the directionality of the effects between LpMTG and LIFG (Cheung et al., in preparation). This analysis showed that the LpMTG drives activity in the LIFG within the 1–20 Hz frequency bands. However, since recurrent activity between regions is an ubiquitous part of network function (Friston, 2003), we anticipate that the normal functioning of the fronto-temporal network includes repeated, recurrent activity between LIFG and LMTG. This may function as background activity as speech is heard and processed, and what we see here is the modulation of this system in cases of sentential ambiguity that must be resolved in order that the listener can compute a coherent representation of an utterance. In future studies we hope to investigate these and related issues in greater detail.

In summary, this study aimed to characterize the syntactic computations that occur within the LIFG and LpMTG core language network as spoken sentences are heard and processed, and the relationship between them. We focused on syntactic ambiguity since it is a normal and frequent aspect of English and, we would argue, invokes the kinds of processes that are routinely used as we seamlessly construct representations of spoken language. However, further studies will need to determine whether the effects we have observed here do indeed generalize to other kinds of syntactic analysis.

## Conflict of Interest Statement

The authors declare that the research was conducted in the absence of any commercial or financial relationships that could be construed as a potential conflict of interest.

## Appendix

**Table A1:** **The 175 sentences**.

Condition	Disambig word	Sentence
Dom	Are	The newspaper reported that accelerating motorbikes are becoming a nuisance
Dom	Are	In warfare, advancing armies are destroying small villages
Dom	Are	The manager explained that advertising awards are presented at fancy ceremonies
Dom	Are	There are many reasons why boiling liquids are to be handled carefully
Dom	Are	The developer knew that building services are supplied by the local council
Dom	Are	At cocktail parties, charming ladies are attractive to older men
Dom	Are	In the long run, cheating partners are likely to get caught
Dom	Are	Early in the morning, clinking bottles are annoying to neighbors
Dom	Are	The class observed that cooking apples are inedible without lots of sugar
Dom	Are	She learnt that cutting boards are easily broken
Dom	Are	On the battlefield, exploding bombs are directed behind enemy lines
Dom	Are	Most experts agree that failing students are not to be rewarded
Dom	Are	After an accident, flashing signs are usually distracting
Dom	Are	Early in summer, growing flowers are weeded regularly
Dom	Are	The teacher was convinced that interesting students are given the most attention
Dom	Are	At demonstrations, irritating policemen are a common sight
Dom	Are	Tom noticed that landing planes are deafening lots of people
Dom	Are	At first, managing assistants are useless without extensive training
Dom	Are	On most roads, passing trucks are a nuisance to other vehicles
Dom	Are	The policeman knew that racing cars are banned on public roads
Dom	Are	On narrow roads, reversing lorries are a problem for other road users
Dom	Are	The teacher explained that rhyming words are found at the end of each line
Dom	Are	The gardener explained that ripening tomatoes are watered daily
Dom	Are	In stormy weather, sailing boats are tossed about on the waves
Dom	Are	On the parade ground, saluting officers are acknowledged by their men
Dom	Are	Captains know that sinking submarines are heading down to the seabed
Dom	Are	In a quiet room, stimulating conversations are a big distraction
Dom	Are	It is important that training athletes are given the correct diet
Dom	Are	Fortunately, understanding parents are common nowadays
Dom	Are	Some teenagers think that worrying parents are a pain in the neck
Dom	Is	On some housing estates, abusing teenagers is perpetrated by rival gangs
Dom	Is	At night attacking strangers is common in the city
Dom	Is	She had heard that breeding pigeons is very popular
Dom	Is	The woman discovered that capsizing canoes is not difficult in the rapids
Dom	Is	In the afternoon, chasing dogs is favored by the naughty children
Dom	Is	Even today conquering countries is an impossible thing to justify
Dom	Is	It is accepted that crashing vehicles is likely to have serious consequences
Dom	Is	Normally, disturbing plans is a tactic to delay things
Dom	Is	Her brother told her that drowning kittens is extremely immoral
Dom	Is	Outdoors, flying kites is a superb way to entertain the children
Dom	Is	Not surprisingly, hunting eagles is banned across Europe
Dom	Is	Experts agree that inspiring youngsters is certain to improve their chances
Dom	Is	Understandably, insulting neighbors is not encouraged
Dom	Is	In the circus, juggling knives is less dangerous than eating fire
Dom	Is	In some countries, kicking donkeys is a serious offense
Dom	Is	She remarked that mocking boyfriends is more trouble than she expected
Dom	Is	She soon learnt that packing cases is quicker than washing clothes
Dom	Is	He realized that parking vans is not encouraged in busy roads
Dom	Is	The cook explained that pickling onions is a way of preserving them
Dom	Is	Everyone knows that playing cards is an excellent way to pass the time
Dom	Is	As a rule, provoking suggestions is what TV presenters try to do
Dom	Is	The magazine said that roasting potatoes is traditional for Sunday lunch
Dom	Is	She told him that shrugging shoulders is an easy way to be rude
Dom	Is	Friends heard that slamming doors is not allowed in John’s house
Dom	Is	Some people believe that spinning coins is more likely to get the attention of the bartender
Dom	Is	The presenter argued that trusting adolescents is not advisable for their teachers
Dom	Is	It’s a fact that waking babies is usually tricky
Dom	Is	Owners will tell you that walking dogs is a great way to get fit
Sub	Are	On some housing estates, abusing teenagers are expected to go for counseling
Sub	Are	It was pointed out that appointing organizations are required to ask for references
Sub	Are	At night, attacking strangers are not to be approached
Sub	Are	She had heard that breeding pigeons are very noisy
Sub	Are	The woman discovered that capsizing canoes are very difficult to turn upright
Sub	Are	In the afternoon, chasing dogs are barking at the frightened cats
Sub	Are	Even today, conquering countries are threatening innocent civilians
Sub	Are	It is accepted that crashing vehicles are likely to hit other vehicles
Sub	Are	Normally, disturbing plans are shelved by senior managers
Sub	Are	Her brother told her that drowning kittens are seldom rescued
Sub	Are	Outdoors, flying kites are exciting when they are soaring
Sub	Are	Not surprisingly, hunting eagles are spotted over mountains
Sub	Are	Experts agree that inspiring youngsters are certain to encourage their friends
Sub	Are	Understandably, insulting neighbors are not respected
Sub	Are	In the circus, juggling knives are less sharp than people think
Sub	Are	In some countries, kicking donkeys are badly beaten
Sub	Are	She remarked that mocking boyfriends are an embarrassment to their girlfriends
Sub	Are	She soon learnt that packing cases are heavier than shopping bags
Sub	Are	The cook explained that pickling onions are sold in the supermarket
Sub	Are	Everyone knows that playing cards are shiny when they are new
Sub	Are	As a rule, provoking suggestions are necessary for lively debate
Sub	Are	The magazine said that roasting potatoes are tastiest with olive oil
Sub	Are	She told him that shrugging shoulders are a sign of boredom
Sub	Are	Friends heard that slamming doors are annoying the neighbors next door
Sub	Are	Some people believe that spinning coins are more likely to land on heads than tails
Sub	Are	The presenter argued that trusting adolescents are very likely to become volunteers
Sub	Are	It’s a fact that waking babies are usually hungry
Sub	Are	Owners will tell you that walking dogs are usually well-trained
Sub	Is	The newspaper reported that accelerating motorbikes is dangerous in the rain
Sub	Is	In warfare, advancing armies is a difficult thing to achieve
Sub	Is	The manager explained that advertising awards is the responsibility of the publicity department
Sub	Is	There are many reasons why boiling liquids is an effective way to kill germs
Sub	Is	The developer knew that building services is part of a successful project
Sub	Is	The newspaper reported that bullying teenagers is bad for their self esteem
Sub	Is	At cocktail parties, charming ladies is what single men like to do
Sub	Is	In the long run, cheating partners is likely to lead to divorce
Sub	Is	His mum thought that cleaning brushes is important after painting
Sub	Is	Early in the morning, clinking bottles is inconsiderate to neighbors
Sub	Is	The class observed that cooking apples is an easy task
Sub	Is	She learnt that cutting boards is a specialist job
Sub	Is	On the battlefield, exploding bombs is a delicate lifesaving procedure
Sub	Is	Most experts agree that failing students is difficult for lecturers
Sub	Is	After an accident, flashing signs is a excellent way to get noticed
Sub	Is	Early in summer, growing flowers is a great pleasure
Sub	Is	The teacher was convinced that interesting students is an important part of teaching
Sub	Is	At demonstrations, irritating policemen is a dangerous thing
Sub	Is	Tom noticed that landing planes is frightening for some pilots
Sub	Is	At first, managing assistants is a rewarding task
Sub	Is	On most roads, passing trucks is impossible in small cars
Sub	Is	The policeman knew that racing cars is illegal along the seafront
Sub	Is	On narrow roads, reversing lorries is difficult for new drivers
Sub	Is	The teacher explained that rhyming words is a standard poetic technique
Sub	Is	The gardener explained that ripening tomatoes is a tricky business
Sub	Is	In stormy weather, sailing boats is really difficult
Sub	Is	On the parade ground, saluting officers is important for discipline
Sub	Is	Captains know that sinking submarines is nearly impossible
Sub	Is	In a quiet room, stimulating conversations is often difficult
Sub	Is	It is important that training athletes is a top priority for schools
Sub	Is	Fortunately, understanding parents is easy today
Sub	Is	Some teenagers think that worrying parents is an acceptable way to behave
Unamb	Are	Her mother told her that crying babies are usually hungry
Unamb	Are	Few teenagers think that living poets are more likely to get the attention of young people
Unamb	Are	Understandably, yawning audiences are not welcomed
Unamb	Are	Even in the daylight, chuckling ghosts are frightening to infants
Unamb	Are	At school, sneering boys are not to be tolerated
Unamb	Are	The head teacher was told that reading problems are really hard to correct
Unamb	Are	Secretaries will tell you that functioning computers are usually reliable
Unamb	Are	The woman knew that glowing references are necessary for the best jobs
Unamb	Are	Outdoors, marching soldiers are frightening when they are noisy
Unamb	Are	On the promenade, joking grannies are heading toward the donkey ride
Unamb	Are	In difficult times, rising costs are a cause of many bankruptcies
Unamb	Are	The assistant knew that discerning consumers are tempted by the latest fashions
Unamb	Are	In the pub, joking comedians are more entertaining than customers expect
Unamb	Are	In the morning, speeding taxis are rushing to the railway station
Unamb	Are	People know that differing views are acceptable these days
Unamb	Are	Not surprisingly, quarreling sisters are sent to bed
Unamb	Are	As a rule, flowering trees are perfect for bigger gardens
Unamb	Are	The government knew that working mothers are happiest with short hours
Unamb	Are	It’s a fact that emerging economies are slowly developing
Unamb	Are	Normally, glistening bracelets are displayed in the shop window
Unamb	Are	It’s obvious that clinging children are lacking some reassurance
Unamb	Are	The employees believe that interfering bosses are a hindrance to their work
Unamb	Are	He told her that aching legs are a problem for runners
Unamb	Are	Late in the evening, laughing friends are shrieking loudly
Unamb	Are	She learnt that traveling businessmen are very pushy
Unamb	Are	Parents believe that camping trips are helping children develop
Unamb	Are	In difficult conditions struggling swimmers are rounded up by the lifeguard
Unamb	Are	She knew that despairing friends are often neglected
Unamb	Is	The article explained that describing paintings is encouraged in the gallery
Unamb	Is	The judge argued that imprisoning thieves is a way of punishing them
Unamb	Is	In some big gardens, preventing weeds is achieved with toxic chemicals
Unamb	Is	There are many reasons why torturing prisoners is an unsatisfactory way to get information
Unamb	Is	The teacher was sure that allowing games is a bad idea in the rain
Unamb	Is	In the long run, employing craftsmen is likely to get the job done
Unamb	Is	In cricket, bribing umpires is a foolish thing
Unamb	Is	At first, establishing friendships is an interesting undertaking
Unamb	Is	In most families, resolving quarrels is nearly impossible
Unamb	Is	Workers understand that neglecting risks is a terrible way to carry on
Unamb	Is	Most experts agree that exploiting schoolchildren is upsetting for parents
Unamb	Is	In most companies, impressing employers is essential to ambitious staff
Unamb	Is	The teacher knew that rehearsing plays is necessary for a good performance
Unamb	Is	Criminals know that regretting crimes is sure to shorten their sentence
Unamb	Is	At Christmas parties, bringing presents is what thoughtful people like to do
Unamb	Is	At the art auction, selecting paintings is fun for everyone
Unamb	Is	The chairperson announced that adopting children is the topic of this week’s debate
Unamb	Is	After redundancy, acquiring debts is a terrible way to get money
Unamb	Is	In some countries, denouncing traitors is a patriotic duty
Unamb	Is	The secretary learnt that shredding files is a standard requirement
Unamb	Is	The headmaster commented that marking essays is a daunting task
Unamb	Is	He found that installing lights is not easy in early February
Unamb	Is	She soon realized that renting flats is cheaper than buying houses
Unamb	Is	It is accepted that releasing terrorists is likely to enrage their victims
Unamb	Is	The gambler told him that predicting results is the only way to make money
Unamb	Is	For young people inheriting fortunes is a terrible burden
Unamb	Is	The nurse explained that bandaging wounds is an important first aid procedure
Unamb	Is	Children know that harming animals is extremely bad
Unamb	Is	The reporter discovered that blaming universities is not fair on the lecturers

*Note that only the sentence fragments up to the disambiguating word were presented in the MEG experiment*.
